# No Overt Effects of a 6-Week Exergame Training on Sensorimotor and Cognitive Function in Older Adults. A Preliminary Investigation

**DOI:** 10.3389/fnhum.2017.00160

**Published:** 2017-04-04

**Authors:** Madeleine Ordnung, Maike Hoff, Elisabeth Kaminski, Arno Villringer, Patrick Ragert

**Affiliations:** ^1^Department of Neurology, Max Planck Institute for Human Cognitive and Brain SciencesLeipzig, Germany; ^2^Mind and Brain Institute, Charité and Humboldt UniversityBerlin, Germany; ^3^Institute for General Kinesiology and Exercise Science, University of LeipzigLeipzig, Germany

**Keywords:** exergames, healthy aging, sensorimotor, cognition, video games, aerobic fitness, motor skills

## Abstract

Several studies investigating the relationship between physical activity and cognition showed that exercise interventions might have beneficial effects on working memory, executive functions as well as motor fitness in old adults. Recently, movement based video games (exergames) have been introduced to have the capability to improve cognitive function in older adults. Healthy aging is associated with a loss of cognitive, as well as sensorimotor functions. During exergaming, participants are required to perform physical activities while being simultaneously surrounded by a cognitively challenging environment. However, only little is known about the impact of exergame training interventions on a broad range of motor, sensory, and cognitive skills. Therefore, the present study aims at investigating the effects of an exergame training over 6 weeks on cognitive, motor, and sensory functions in healthy old participants. For this purpose, 30 neurologically healthy older adults were randomly assigned to either an experimental (ETG, *n* = 15, 1 h training, twice a week) or a control group (NTG, *n* = 15, no training). Several cognitive tests were performed before and after exergaming in order to capture potential training-induced effects on processing speed as well as on executive functions. To measure the impact of exergaming on sensorimotor performance, a test battery consisting of pinch and grip force of the hand, tactile acuity, eye-hand coordination, flexibility, reaction time, coordination, and static balance were additionally performed. While we observed significant improvements in the trained exergame (mainly in tasks that required a high load of coordinative abilities), these gains did not result in differential performance improvements when comparing ETG and NTG. The only exergaming-induced difference was a superior behavioral gain in fine motor skills of the left hand in ETG compared to NTG. In an exploratory analysis, within-group comparison revealed improvements in sensorimotor and cognitive tasks (ETG) while NTG only showed an improvement in a static balance test. Taken together, the present study indicates that even though exergames might improve gaming performance, our behavioral assessment was probably not sensitive enough to capture exergaming-induced improvements. Hence, we suggest to use more tailored outcome measures in future studies to assess potential exergaming-induced changes.

## Introduction

In the last decades, great effort has been put into understanding how to improve healthy and successful aging, since a prolonged lifespan has major implications for our health care and social system.

Typically, aging is accompanied with a decline in cognitive (Park et al., 2002; Verhaeghen and Cerella, 2002; Salthouse, 2004) and sensorimotor functions (Stevens and Choo, 1996; Konrad et al., 1999; Smith et al., 1999; Krampe, 2002; Li and Lindenberger, 2002). However, there are substantial individual differences in how much people are affected by age-related decline. And it seems that some people are relatively spared of age-related alterations and disabilities (Nelson and Dannefer, 1992; Morse, 1993; Christensen et al., 1994; Fozard et al., 1994; Stewart et al., 2014). In this regard, successful aging has become a familiar term and is defined by three components: being actively engaged in life, the absence of disease as well as physical and mental fitness (Rowe and Kahn, 1997). More importantly, these components are not acting independently; they rather contribute in an interrelated fashion. Accordingly, many studies emphasized the key role of physical activities in preventing as inevitable seeming age-related chronic diseases, neurodegenerative, and psychiatric disorders (Sacco et al., 1998; Franklin et al., 2001; Strawbridge et al., 2002; Ravaglia et al., 2008; Ciolac, 2013). Being engaged in physical activity can further contribute significantly to life satisfaction and happiness in later life (Menec, 2003; Liffiton et al., 2012). Moreover, there is compelling evidence that exercising is an effective approach to prevent cognitive decline associated with increasing age (Hertzog et al., 2008; Bherer et al., 2013). For instance, it is well established that aerobic training not only improves motor fitness, it also positively influences cognitive performance, such as working memory and executive functions. Furthermore, these behavioral benefits are accompanied by structural and functional brain adaptations (Kramer et al., 1999; Colcombe et al., 2006; Angevaren et al., 2008; Erickson et al., 2011; Voelcker-Rehage et al., 2011; Voss et al., 2013; Bamidis et al., 2014), like volume changes in cortical motor and frontal areas as well as in subcortical structures. Interestingly, these changes are not exclusively induced by cardiovascular training. Coordination training combining balance, limb coordination, spatial orientation and reaction time tasks are also effective in modulating brain structure and function that are associated with improvements in cognition and motor performance (Voelcker-Rehage et al., 2010, 2011). Comparative studies revealed that combining motor and cognitive demands during exercising can even lead to greater enhancements in cognition than training the domains separately (Fabre et al., 2002; Oswald et al., 2006; Lauenroth et al., 2016). It has been argued that enhanced neuronal metabolic processes induced by physical activity can only be exploited efficiently when the brain is simultaneously challenged by cognitive demands (Oswald et al., 2006; Bamidis et al., 2014). Moreover, it is thought that multi-modal training interventions resemble real-life demands and therefore yield higher chances of successful transfer to other tasks and every-day life situations (Lustig et al., 2009). In line with this, recent studies have highlighted the capacity of lifestyle interventions, like dancing (Kattenstroth et al., 2013; Coubard et al., 2014) or playing video games (Maillot et al., 2012; Pichierri et al., 2012), to enhance sensorimotor and cognitive functions in older adults. Investigating the potential of video games in preventing age-related cognitive decline has gained great research interest within the last years (Green and Seitz, 2015). It has been frequently shown that cognitive video game training can have beneficial effects on several cognitive functions in older adults, including memory, attention, and reaction time (Lampit et al., 2014; Toril et al., 2014). While classical video games being played sedentarily, movement based video games (exergames) require the performance of physical activities while being simultaneously surrounded by a cognitively challenging environment. Studies were able to show that exergaming for instance can promote executive functions and cognitive processing speed in older adults (Maillot et al., 2012; Schoene et al., 2013). Furthermore, a recent review article could show that various studies using exergame trainings, show beneficial effects on cognitive, as well as on dual-task performance, which potentially reduces the risk of falls in older adults (Ogawa et al., 2016). Nevertheless, exergame intervention studies attempting to examine effects on motor performance, investigated mainly the effect on balance, which showed rather mixed results (Bisson et al., 2007; Nicholson et al., 2015). The same holds true for improvements in muscle strength of lower and upper limbs (Nitz et al., 2010; Jorgensen et al., 2013). Moreover, benefits are mostly shown for trained tasks but only limited transfer effects to other tests (Bisson et al., 2007; Pichierri et al., 2012; Baniqued et al., 2014; Sato et al., 2014). Video game characteristics might contribute to this since most of the video games used are tailor-made, representing predominantly highly controlled laboratory conditions and therefore missing a multi-modal environment promoting transfer (Lustig et al., 2009; Baniqued et al., 2014). In contrast, commercial video games which, for instance, include different sport disciplines, require fast reactions and the ability to shift the attentional focus for performing appropriate and well timed movements. Up do date, studies examining commercial video games were mostly conducted using different applications of Nintendo®, for instance Nintendo Wii™ (Nintendo Co. Ltd., Kyoto, Japan). This system is equipped with a hand remote controller which requires to play the game in performing physical gestures by moving upper limbs. A more recent development are consoles using a camera system, for instance Xbox™ 360 Kinect™ (Microsoft corp., Redmond, WA), which recognizes gestures and therefore requires whole body movements for playing (van Diest et al., 2013). Up to now, only a few studies examined the effects of a whole-body exergame training and they have indicated improvements in balance (van Diest et al., 2016) and lower extremity muscle strength (Sato et al., 2014). In fact, to date little is known about the impact of a whole-body and multi-modal exergame training intervention on a broad range of sensorimotor and cognitive skills, such as aerobic fitness, fine motor skills, tactile acuity, and working memory. Furthermore, sample characteristics between studies differ to a great extent and ranging from hospitalized, community dwelling (Jorgensen et al., 2013; Schoene et al., 2013; Sato et al., 2014; Nicholson et al., 2015; van Diest et al., 2016) to independent living sedentary participants (Maillot et al., 2012). Until now, it is not known whether healthy active older adults would also benefit from exergame training. Likewise, there is a huge difference in study design of previously performed studies. They differ in duration and frequency of the training program and the standardization of the setting (home-based or under supervision in the lab; Maillot et al., 2012; Schoene et al., 2013; van Diest et al., 2016). Typically, studies so far were taken place within training periods up to 12 weeks, with a training frequency of 2–3 sessions per week (Hall et al., 2012; Marston and Smith, 2012). Interestingly, Colcombe and Kramer (2003) stated that effects of physical training on cognition can already be found after 1 month of training, while longer training periods show substantially larger effects. Therefore, the current study aimed to investigate the effect of a whole-body exergame training intervention using the console Xbox™ 360 Kinect™ over a rather short training period of 6 weeks. Based on previous findings from the aforementioned studies, we expected that a multi-domain video exergame training combining endurance, coordination, strength as well as demands on cognitive processing will translate into overall, and therefore generalized enhancements of a broad range of sensorimotor as well as cognitive functions. Following on this, we further wanted to examine the potential benefits of exergame training for provoking benefits in a healthy, active sample of older adults. We therefore hypothesized that exergaming will induce significant improvements in the performance of sensorimotor and cognitive functions compared with a group receiving no training. We further expected that the exergame training group will show significant practice effects during exergaming and that these online improvements will be associated with the baseline performance in the sensorimotor and cognitive tests.

## Materials and methods

### Participants

A total number of 30 healthy participants were enrolled in the present study after giving written informed consent. To exclude any evidence for neurological disease and contraindications with respect to the study procedures, all participants were neurologically examined by a physician. All participants were free of taking any central-acting drugs and completed the Mini-Mental State Examination (MMSE; Folstein et al., 1983). We further did not include highly skilled musicians, typists, or sportsmen. Nevertheless, some of the participants were experienced in playing a musical instrument or did sports on a regular basis as assessed by a questionnaire (for group characteristics also see Table 1). Handedness was assessed using the Edinburgh Handedness Inventory (Oldfield, 1971). According to this questionnaire, three participants were considered ambidextrous (mean score: 2; range −10–26) and therefore excluded for the analysis on hand function, grip strength and pinch force, as well as touch sensitivity. The remaining participants were all right handed (91.81; range 70–100). None of the participants reported to have experiences in playing exergames. The participants were randomly assigned to a passive no training group (*n* = 15, 8 female, mean age: 68.6 ± 4.67) or the exergame training group (*n* = 14, 7 female, mean age: 69.79 ± 6.34). All subjects gave written informed consent in accordance with the declaration of Helsinki. The protocol was approved by the local ethics committee of the University of Leipzig (ref no. 376-15-24082015).

**Table 1:** **Group demographics**.

**Variable**	**ETG**		**NTG**		
	**M**	**SD**	**M**	**SD**	***p*-value**
Age	69.79	6.34	68.6	4.67	0.569
Education	1.93	0.83	1.47	0.74	0.125
MMSE	29.36	1.22	28.73	1.1	0.158
BMI	25.54	3.84	26.36	4.85	0.665
Physical activity	1.64	1.22	1.73	2.22	0.894
Musical training	0.21	0.43	0.33	0.49	0.491

*Education: years of school; total score range 1–3 (1 = at least 12 years; 2 = 10 years, 3 = 8 years). MMSE, Mini Mental State Examination; total score range of 1–30; cut-off score for exclusion: <26. BMI, Body Mass Index; Physical Activity: average hours of sport activity per week. Music training: average hours of playing a musical instrument. All values are depicted as mean ± standard deviation of the mean. ETG, Exergame Training Group; NTG, No Training Group*.

### Experimental procedure

**Figure 1:**
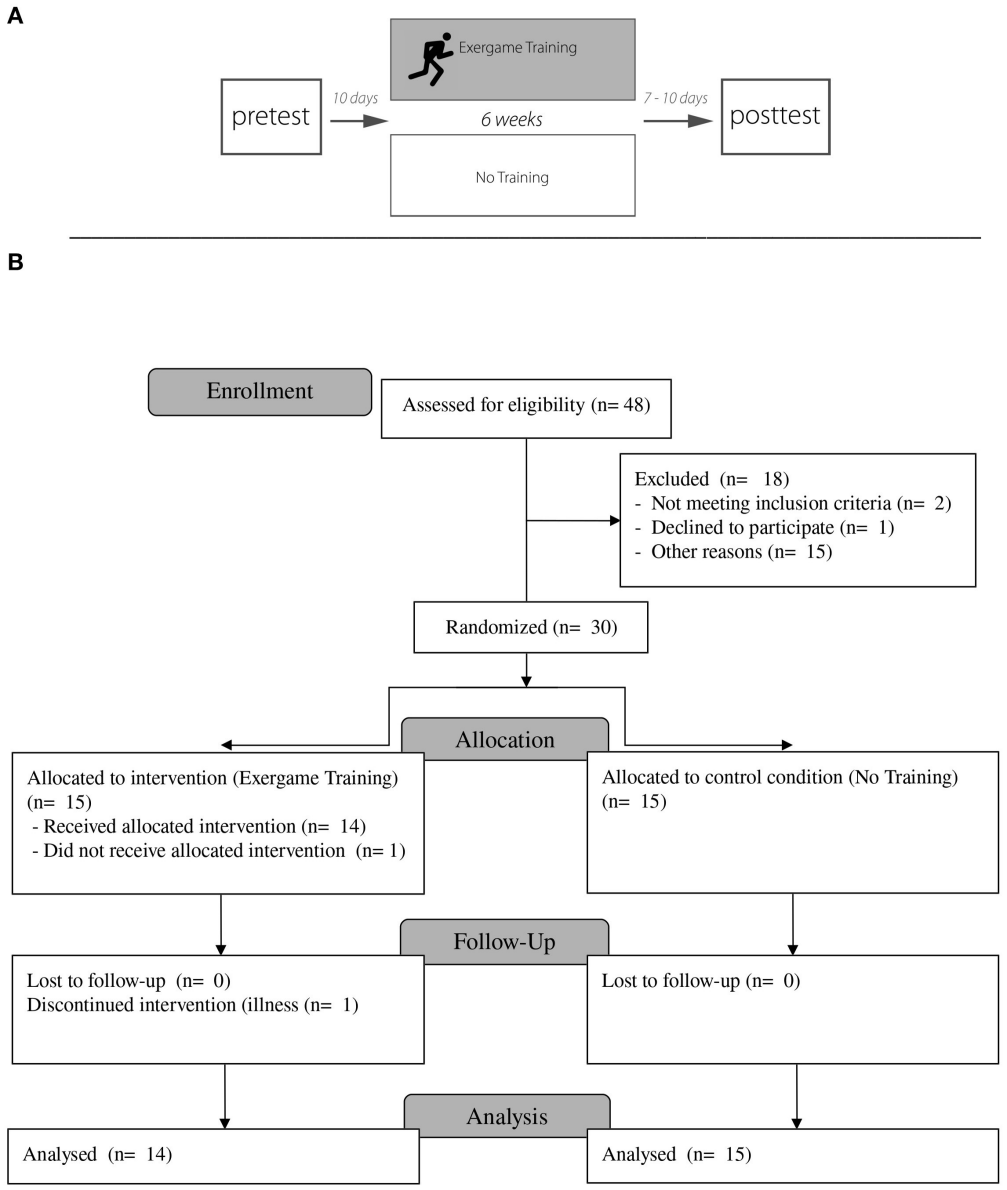
**Experimental setup and design. (A)** Subjects were randomly assigned to a intervention group (exergame training), participating in a exergame training over 6 weeks, or a passive control group (no training group). All participants completed cognitive as well as sensorimotor tests 10 days before (pretest) the intervention. The same tests were administered with cognitive testings 10 days and sensorimotor testings 7 days after the intervention. **(B)** Flow diagram presenting the enrollment, intervention allocation, follow-up, and data analysis with the respective number of participants.

We used a between groups, pretest-training-posttest design for which participants were randomly assigned to either the exergame training group (ETG) or a passive no training group (NTG). All participants completed cognitive tests as well as sensorimotor tests before and after the respective intervention (see also Figure 1). The tests were administered each time in a random order over 2 days. Prior to the pre and post-tests for both groups as well as before each session for the ETG, level of attention, fatigue and discomfort was examined using a visual analog scale (VAS) ranging from 1 to 10 (1 = very inattentive to 10 = very attentive; 1 = high fatigue to 10 low fatigue; 1 = no discomfort to 10 = high discomfort). For the ETG, motivation to train was administered at the beginning of each training session (1 = very unmotivated to 10 = very motivated). Between pre and post assessments, participants from the ETG performed in total 12 exergame training sessions over 6 weeks, with 2 sessions per week. Each session lasted 60 min resulting in a total training time of 12 h. For avoiding potential influences by social interactions, each participant received personal training sessions. At the beginning of each session pulse at rest was measured using a pulse oximeter, placed at the index finger. In order to reduce the risk of injuries a standardized warm-up of 5 min was performed prior to the exergame training.

### Outcome variables

#### Aerobic fitness and sensorimotor performance

##### Aerobic fitness

To characterize aerobic fitness, we used the 3 Min Step Test (ACSM, 2010) measuring pulse recovery to a functional activity (step climbing). The test was performed on an aerobic platform (heights: 24 cm) and step cadence was controlled using a metronome which was set to 84 beats per minute (bpm). Prior to the test, resting pulse (RP) on the index finger was measured with a finger-tip pulse oximeter (PULOX® PO-200, Novidion GmbH, Germany). Following the demonstration by an instructor, the participants performed the test for 3 min. Immediately after finishing the test, strained pulse (ST) and 1 min later recovery pulse (ReP) were measured. For the analysis we computed the Ruffier performance index (Rodríguez Cabrero, 2015) using the following formula: [(RP + ST + ReP) − 200]/10. Participants were excluded from the analysis if they could not keep the given cadence or if they had to stop the test due to exhaustion. For those reasons, data of three participants had to be excluded from the analysis.

##### Upper body muscular endurance

For measuring muscular endurance of the upper body, participants were asked to lie prone on an exercise mattress with the forehead resting on a wedge pillow. The participants were given a dumbbell in each hand and were instructed to perform a rowing motion simultaneously with both arms (stretching and bending of the arms) between two markers. The movement frequency was determined by a metronome which was set to 60 bpm. Upper arms and elbows had to form an angle of 90°, slightly touching a marker on the back. For performing the stretching movement, the hands with the dumbbells were supposed to touch the front marker. The number of movement repetitions served as outcome measurement. The test was stopped if the participants were touching the mattress with the dumbbells, if they could not keep the movement frequency or if they were not able to touch the two markers (front or back) anymore. Female participants had to move dumbbells with a weight of 1 kg each; males were tested using 2 kg dumbbells. Data of two participants could not be collected due to non-attendance.

##### Grip strength

The maximum strength of the hand and forearm muscle was analyzed using a hydraulic dynamometer (SEAHAN®, SEAHAN Corporation, S. Korea). Participants sat in an upright position and were instructed to hold the dynamometer in the hand while keeping the elbow by the side of the body and the arm at right angles. For the testing, participants had to squeeze the dynamometer for 5 s applying maximum isometric effort. Left and right hand were measured three times in a randomized order. Grip strength was measured in kg and for the final analysis mean performance of each side was used. Due to non-attendance, data from one participant could not be collected.

##### Pinch force

Maximum pinch force of the thumb was evaluated using a hydraulic pinch gauge (SEAHAN®, SEAHAN Corporation, S. Korea). Participants were asked to take an upright position with the arm forming an angle of 90° while keeping the elbow on the side. Participants were holding the pinch gauge between the proximal interphalangeal joint of the index finger and the thumb. The pinch gauge had to be squeezed with maximum force three times each side. Each trial lasted 5 s with a break of 10 s in between. The dependent variable was mean performance of each side in kg.

##### Motor reaction time

For measuring motor reaction time we used the Ruler Drop Test (Del Rossi et al., 2014). Participants were seated sideways on a chair with the forearm placed over the edge of the chair. The bottom of the measuring stick was placed perpendicular between the thumb and the index finger of the participant. After an acoustic warning signal, the stick was dropped and the participant had to catch it as fast as possible. We recorded the number on the measuring stick displayed just over the thumb representing the reaction time. The test was performed with the dominant hand. Due to non-attendance, data from one participant could not be collected.

##### Hand motor skills

Fine motor skills of the upper extremities were evaluated using the Jebson-Taylor Hand Function Test (JTT) (Jebsen et al., 1969). Originally the JTT consists of seven different subtests from which six were included in this study: turning over cards, picking up small objects and placing them in a can, picking up small objects with a teaspoon and placing them in a can, stacking chequers, moving large light-weighted cans, and moving heavy-weighted cans. Participants were instructed to perform the tasks as fast and accurate as possible. All subtests were performed separately with left and right hand with left-hand performance always evaluated first. For the analysis, we recorded times for completion of each task and each hand.

##### Flexibility

Flexibility was characterized using the Back Scratch Test (Konopack et al., 2008) which was performed in a sitting position. Participants had to place one hand behind the head and back over the shoulder and reaching down the back as far as possible. The palm was supposed to touch the body and the fingers were directed downwards. The other arm was also moved behind the back under the shoulder with the palm turned outward and fingers upward reaching the fingers from the other hand. The movements of the arms had to be performed without any momentum. As the dependent variable, distance or overlap in centimeter from the two middle fingers was assessed. If there was a distance between the fingers, participants received a negative score while, for overlapping fingers, a positive score was attributed. If the fingertips were exactly touching, the given score was zero. Due to non-attendance data from one participant could not be collected.

##### Static balance

For investigating static balance we used the Wii Balanceboard© (Nintendo Co. Ltd., Kyoto, Japan) which has been shown to reliable measure balance in the elderly (Chang et al., 2013). Static stance was assessed under four different conditions: standing still on both legs eyes open and closed, on the right leg and left leg eyes open. Each condition lasted for 15 s. Subjects were instructed to stand as quietly as possible on the platform. Prior the testing, individual center of pressure was calibrated. For all conditions, the average body sway in anterior-posterior (AP) and medio-lateral (ML) direction was analyzed using the STABLE software from pro-WISS© (Bochum, Germany).

##### Tactile performance

Tactile performance was measured using Touch-Test™ Sensory Evaluators (Semmes-Weinstein Monofilaments, North Coast Medical, Inc., CA). The test kit contained 20 filaments differing in length and diameter, therefore resulting in a specific buckling force. Hence, the applied forces were ranging between 0.008 and 300 mN. The filaments were pressed in a 90° angle against the tip of the index finger until they bowed and were hold in place for approximately 2 s. The participants were asked to close their eyes and respond as soon as the stimulus was felt. The test was performed using a staircase procedure starting with the filament representing the highest force by decreasing until the participant could not perceive the stimulus anymore (lower boundary). The forces then were increased until the participant reported an indentation again (upper boundary). In order to receive the absolute touch threshold, this procedure was repeated for three times and the resulting six values were averaged.

#### Cognition

Cognitive performance was measured using subtests from the TAP 2.3 (Test of Attentional Performance, PsyTest, Herzogenrath), a computer-based neuropsychological test-battery for analyzing different aspects of attention (Zimmermann and Fimm, 1995). Therefore, the participants were seated in front of a computer screen with hands placed in front of two response keys (left and right). For familiarizing the participants with each subtest, a short exercise session followed the instructions. Subtests were presented in randomized order.

##### Alertness and simple reaction time

Reaction time was measured under two conditions. The first condition concerned simple reaction time to a visual stimuli (greek cross) appearing in random intervals on the screen. Participants had to respond as quickly as possible by pressing a key. In the second condition, the visual stimuli was preceded by a cue in form of a warning tone. The cross followed the warning tone in random intervals. Mean reaction times in ms for both conditions were assessed.

##### Working memory

Working memory capacity and information flow was evaluated by using a N-back task (*N* = 2). Therefore, a sequence of numbers was presented on the screen. Participants were instructed to press the key if the given number was the same as the last but one number. We assessed the number of correct trials out of 15 in 5 min. Due to technical problems, data of one participant was discarded for the final analysis.

##### Response inhibition

This paradigm was used to measure the participant's capability to inhibit a response triggered by an external stimulus. For this either an upright cross (Go-stimulus) or a diagonal cross (NoGo-stimulus) was presented on the screen. Participants were instructed to press a key as fast as possible only when the Go-stimulus appeared. The average of correct answers out of 20 in 2 min was used as dependent variable.

### Exergame training

For the training we used the Microsoft X box 360™, a commercial video game console, in combination with the Microsoft Kinect Sensor™. The Kinect sensor is a horizontal bar which is connected to a motorized base. Equipped with a RGB camera, a depth sensor and a multi-array microphone the Kinect sensor provides full-body 3D motion capture. Hence, without any need for a game controller the sensor enables exergaming via whole body movements. Connected to a screen the Kinect sensor was placed in 2 ms distance frontal to the participants. While training, the Kinect sensor was tracking the participants' movements and therefore allowed them to control their avatars, a graphical representation of the user, in the game. Every training session included different sport games from the commercial video game *Summer Stars 2012* (DEEP SILVER GmbH, Munich Germany). Based on their primarily required motor proficiency, the games were categorized as endurance (freestyle and butterfly swimming, hammer throwing), strength (hurdles, javelin throwing, 100 meter running), and coordination (trampoline, high diving, archery, mountain biking) disciplines. Each participant was playing two times eight disciplines which were presented in a fixed order. Gaming scores for each discipline and every session were noted to examine practice effects. For motivating adherence and enjoyment, 100 meter running and butterfly swimming were replaced by hurdles and freestyle swimming after 6 training sessions, due to similarly required skills. To prevent overexertion pulse was monitored throughout each training session, assessed before and right after every discipline. Each training session was supervised by an instructor ensuring the participants safety and the correct performance of the required movements. For this purpose participants practiced only half of the training program during the first week of training. The participants in the NTG were instructed to keep their usual lifestyle over the entire time of the study.

### Data analysis

Statistical analyses of the data were performed using the statistical software package for social sciences (IBM SPSS v22), figures were made using RStudio 3.3.1 (RStudio Team, 2015). As an initial step, data-sets were checked for normal distribution using the Kolmogorov-Smirnov Test. Since behavioral data were not normally distributed for some outcome measures, we subsequently performed non-parametric statistical tests to evaluate performance improvements within- and between-groups. Effect-sizes were analyzed accordingly, using effect-size measurement r. Comparing VAS, we performed parametric statistical tests using an rmANOVA with factor TIME × GROUP. Sample demographics were compared using two-sample *t*-Tests. Effect size for ANOVA was reported as $\eta_{p}^{2}$. Effect size for *T*-tests as Cohen's d.

One participant was excluded from the final analysis due to non-attendance of at least 80 percent of all training sessions. Therefore, the data of 29 participants (mean age: 69.17 ± 5.47 years; range 60–78 years; 15 females) were included in the final analysis.

#### Group comparisons of sensorimotor and cognitive performance measures

To unravel potential pre-training differences, we compared the baseline scores from both groups in the sensorimotor and cognitive performance measures using the Mann-Whitney *U*-Test for two independent samples. Based on significant pre-training differences between both groups, we then computed a gain score of each subtest representing the percentage performance improvement using the following formula:

$$\begin{array}{l} {\left[\left(\text{post}-\text{pre}\right)/\text{pre}\right]}^{*}100 \end{array}$$

pre (raw test score before the training period), post (raw test score after the training period).

Thereby, we assured that performance gain was evaluated independent from potential baseline differences between groups. In order to examine offline effects induced by the exergame training, gain scores were subsequently compared between-groups using Mann-Whitney *U*-test for two independent samples.

#### Practice effects and baseline dependency of exergaming performance

For excluding influences on gaming performance, we analyzed the average VAS scores and motivation to train before and after each training session using paired *t*-tests. To characterize online training effects, gain scores (see formula above) out of the noted raw game scores from the first (pre) and last session (post) for each sport discipline were calculated. Statistical evaluation was carried out using one sample Wilcoxon signed rank test. Bonferroni correction was applied if necessary to account for multiple comparisons. Within- and between-group comparisons were assessed at the 5% level of significance.

## Results

### Demographics

According to independent samples *t*-tests, there were no significant between-group differences regarding age [*t*_(27)_ = −0.57, *p* = 0.569, *d* = −0.21], years of education [*t*_(27)_ = −1.58, *p* = 0.125, *d* = −0.59], MMSE scores [*t*_(27)_ = −1.45, *p* = 0.158, *d* = −0.54], body mass index [*t*_(20)_ = 0.44, *p* = 0.665, *d* = 0.19], hours of physical activity [*t*_(27)_ = 0.14, *p* = 0.894, *d* = 0.05] and hours of playing a musical instrument per week [*t*_(27)_ = 0.70, *p* = 0.491, *d* = 0.27] (see also Table 1 for mean values). RM-ANOVA revealed no group differences in fatigue [*F*_(1, 25)_ = 1.23, *p* = 0.279, $\eta_{p}^{2}$ = 0.047] and discomfort [*F*_(1, 25)_ = 0, *p* = 0.946, $\eta_{p}^{2}$ = 0.001] but a significant TIME × GROUP interaction for attention [*F*_(1, 25)_ = 7.17, *p* = 0.013, $\eta_{p}^{2}$ = 0.223]. Nevertheless, considering the mean values (see also Table 2), we believe that there was no impact from attention on the post-performance of the NTG.

**Table 2:** **Visual Analogue Scale (VAS)**.

**Variable**	**ETG**		**NTG**		
	**M**	**SD**	**M**	**SD**	***p*-value**
Attention	8.46	0.77	8.89	1	0.075
Fatigue	8.61	0.84	9.18	1.15	0.065
Discomfort	1.1	0.79	1.03	0.73	0.521

*A visual analog scale was used to assess attention, fatigue and discomfort before the pre-training and post-training measurements. Attention scale ranging from 1 to 10 (1 = very inattentive to 10 = very attentive). Fatigue scale, from 1 to 10 (1 = high fatigue to 10 low fatigue) and discomfort scale ranging from 1 to 10 (1 = no discomfort to 10 = high discomfort). All values are expressed as mean ± standard deviation. ETG, Exergame Training Group; NTG, No Training Group*.

### Group comparisons of sensorimotor and cognitive performance measures

**Figure 2:**
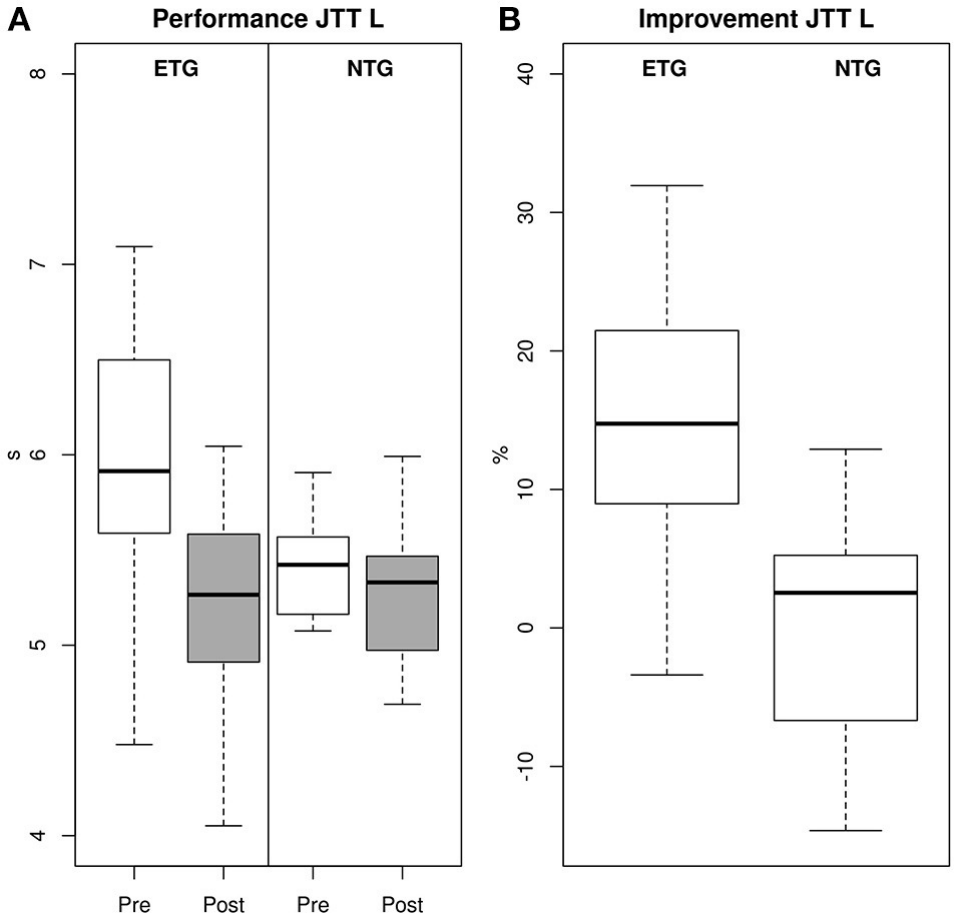
**Exergame training-induced improvements in motor performance**. **(A)** Mean performance in JTT (left hand). There were significant differences at baseline for the JTT (left hand) indicating that both groups started at different performance levels. After the exergame training the ETG were able to complete the JTT faster than the NTG. **(B)** Relative improvement in JTT (left hand). In order to account for baseline differences performance was analyzed independently from baseline measures by calculating a relative gain score. When comparing the relative gains score between both groups, we observed significant improvements for the ETG compare to the NTG.

We found significant between-group baseline differences in JTT performance of the left hand (Mann-Whitney *U*-Test for two independent samples, ETG Mdn: 5.91 s, NTG Mdn: 5.34 s, *U* = −3.275, *p* = 0.001, *r* = 0.61), as well as in the touch test of the right hand (ETG Mdn: 3.32 mN, NTG Mdn: 3.03 mN, *U* = −2.389, *p* = 0.016, *r* = 0.44). Please see Table 3 for details on all variables tested. When comparing the gain scores of both groups, we found significantly greater improvements for the ETG in the JTT performance of the left hand (ETG Mdn: 14.747%, NTG Mdn: 2.538%, *U* = −3.230, *p* = 0.001, see also Figure 2). However, all other variables showed no significant differences between groups. The descriptives and inferentials for all variables are given in Table 4.

**Table 3:** **Baseline characteristics of both groups**.

**Variables**	**ETG**		**NTG**				
	**Median**	**Min–Max**	**Median**	**Min–Max**	**U**	***p*-value**	**Effect-size r**
**AEROBIC FITNESS AND SENSORIMOTOR PERFORMANCE**							
Step test (Index)	10.60	0.0–14.40	7.50	0–18.2	−0.917	0.377	0.17
Upper body muscular endurance test (rep.)	26.00	0.0–40.00	22.00	0–77.0	−0.529	0.621	0.10
Grip strength test R (kg)	21.32	16.53–49.59	27.83	0–48.08	−0.568	0.591	0.11
Grip strength test L (kg)	21.09	16.18–42.03	27.67	0–45.51	−0.371	0.715	0.07
Pinch force test R (kg)	6.16	4.54–12.70	7.12	0–12.4	−0.044	0.983	0.01
Pinch force test L (kg)	6.61	4.55–11.17	6.65	0–12.25	−0.284	0.780	0.05
Motor reaction time test (cm)	17.00	0.00–28.00	17.0	9.50–32.50	−0.459	0.652	0.09
JTT R (s)	4.91	4.38–6.69	4.80	0–5.27	−1.179	0.252	0.22
JTT L (s)	5.91	4.48–8.08	5.34	0–5.91	−3.275	0.001^*^	0.61
Back scratch test R (cm)	−12.50	−30.00–12.00	−2.0	−38.0–12.0	−0.415	0.683	0.08
Back scratch test L (cm)	−16.00	−39.00–6.00	−8.0	−35.0–12.0	−1.026	0.310	0.19
COP AP EO (mps)	3.90	2.10–6.30	4.8	2.90–11.0	−1.719	0.077	0.32
COP AP EC (mps)	7.05	3.20–13.50	6.60	3.0–11.80	−0.175	0.880	0.03
COP ML EO (mps)	2.15	1.00–4.10	1.80	1.10–3.40	−1.116	0.270	0.21
COP ML EC (mps)	2.90	1.30–9.00	2.80	1.10–5.90	−0.459	0.652	0.09
Touch-test R (mN)	3.32	3.09–3.60	3.03	0.0–3.90	−2.389	0.016	0.44
Touch-test L (mN)	3.22	2.43–4.06	3.16	0.0–3.74	−0.918	0.377	0.17
**COGNITION**							
Alertness (ms)	273.75	237.40–390.70	254.0	229.50–330.70	−1.746	0.085	0.32
Simple reaction time (ms)	274.55	230.60–391.30	254.90	227.10–329.60	−1.200	0.234	0.22
Working memory (correct)	9.00	−26.00–15.00	13.0	−1.0–15.0	−1.744	0.085	0.32
Response inhibition (correct)	19.00	10.00–20.00	19.0	16.0–20.0	−1.021	0.331	0.19

*ETG, Exergame Training Group; NTG, No Training Group; Min, Minimum; Max, Maximum; COP, Center of pressure deviation; AP, Anterior-Posterior; ML, medio-lateral; EO, eyes open; EC, eyes closed; U-Test-statistic for Mann-Whitney-U nonparametric test for the comparison of two independent samples, Effect-size r: r = |z/√n|; r = 0.01: small effect, r = 0.30: medium effect; r = 0.50: large effect*.

* *indicate significant finding*.

**Table 4:** **Group comparisons of sensormimotor and cognitive performance gains**.

**Variables**	**ETG**		**NTG**				
	**Median**	**Min–max**	**Median**	**Min–max**	**U**	***p*-value**	**Effect-size r**
**AEROBIC FITNESS AND SENSORIMOTOR PERFORMANCE**							
Step test (Index)	10.047	−52.73–70.00	−2.236	−104.41–45.05	−1.878	0.063	0.35
Upper body muscular endurance test (rep.)	−2.00	−47.06–87.50	−3.226	−81.48–100.00	−0.568	0.591	0.11
Grip strength test R (kg)	2.475	−16.56–19.17	−6.232	−14.03–25.44	−1.615	0.112	0.30
Grip strength test L (kg)	1.159	−32.14–19.50	−8.807	−26.80–9.35	−1.746	0.081	0.32
Pinch force test R (kg)	12.512	−20.74–78.69	18.494	−16.12–58.28	−0.611	0.561	0.11
Pinch force test L (kg)	28.077	−5.13–48.61	17.411	−18.35–42.23	−1.571	0.123	0.29
Motor reaction time test (cm)	0.00	−41.94–23.21	−21.053	−115.79–37.84	0.590	0.561	0.11
JTT R (s)	6.652	0.04–28.87	5.566	−18.00–18.37	−0.567	0.561	0.11
JTT L (s)	14.747	−3.40–31.94	2.538	−14.64–12.90	−3.230	0.001^*^	0.60
Back scratch test R (cm)	−5.556	−110.00–75.00	−5.263	−275.00–500.00	−0.611	0.561	0.11
Back scratch test L (cm)	−3.571	−1900.00–80.00	0.00	−230.00–200.00	−0.655	0.533	0.12
COP AP EO (mps)	−4.021	−121.05–50.00	5.00	−56.25–71.11	−1.135	0.270	0.21
COP AP EC (mps)	0.00	−135.48–60.98	−20.69	−81.82–64.00	−0.131	0.914	0.02
COP ML EO (mps)	23.132	−94.34–50.79	11.11	−6.98–42.37	−0.873	0.400	0.16
COP ML EC (mps)	25.238	−119.35–65.56	7.692	−36.36–65.52	−0.458	0.652	0.09
Touch-test R (mN)	2.104	−13.91–20.60	1.979	−16.67–27.34	−0.306	0.780	0.06
Touch-test L (mN)	3.074	−13.94–11.43	3.881	−14.10–35.44	−0.284	0.780	0.05
**COGNITION**							
Alertness (ms)	4.500	−27.08–25.70	2.026	−32.62–24.97	−1.047	0.310	0.19
Simple reaction time (ms)	10.185	−14.90–32.35	5.398	−15.35–18.97	−1.571	0.123	0.29
Working memory (correct)	0.00	−66–67–200.00	0.00	−400.00–16.67	−1.263	0.217	0.23
Response inhibition (correct)	0.00	−5.26–50.00	0.00	−10.53–18.75	−1.230	0.234	0.23

*ET, Exergame Training Group; NTG, No Training Group; Min, Minimum; Max, Maximum; COP, Center of pressure deviation; AP, Anterior-Posterior; ML, medio-lateral; EO, eyes open; EC, eyes closed; Effect-size r: r = |z/√n|; r = 0.01; small effect; r = 0.30: medium effect; r = 0.50: large effect*.

* *indicate significant finding*.

### Within-group comparisons of sensorimotor and cognitive performance measures

Within-group comparison were done in an exploratory fashion, to investigate potential training effects and their effect sizes, in the ETG and NTG separately. The ETG showed significant performance improvements from baseline to post-measurement in pinch force of both hands (Wilcoxon-Test for two related samples, Pinch Force Test right, Mdn pre: 6.16 kg, Mdn post: 7.40 kg, *W* = −2.542, *p* = 0.011, *r* = 0.68, Pinch Force Test left, Mdn pre: 6.61, Mdn post:6.95, *W* = −2.982, *p* = 0.003, *r* = 0.80), in fine motor function of both hands as assessed with the JTT (JTT right hand Mdn pre: 4.91 s, Mdn post:4.62 s, *W* = −3.296, *p* = 0.00, *r* = 0.88, JTT left hand Mdn pre: 5.91 s. Mdn post: 5.27 s, *W* = −3.107, *p* = 0.002, *r* = 0.83), in the assessment of static balance with eyes closed (COP AP Mdn pre: 7.05 mps, Mdn post: 6.00 mps, *W* = −2.010, *p* = 0.044, *r* = 0.54, COP ML Mdn pre: 2.90 mps, Mdn post: 2.10, *W* = −1.992, *p* = 0.046, *r* = 0.53) and in two cognitive assessments (Alertness Mdn pre: 273.75 ms, Mdn post: 241.10 ms, *W* = −2.291, *p* = 0.022, *r* = 0.61, simple reaction time Mdn pre: 274.55 ms, Mdn post: 251.60 ms, *W* = −2.040, *p* = 0.041, *r* = 0.55). However, the NTG only showed significant performance improvements in one assessment of static balance (COP AP Mdn pre: 6.60 mps, Mdn post: 6.00 mps, *W* = −2.786, *p* = 0.005, *r* = 0.72). Please see Table 5 for details.

**Table 5:** **Within-group comparison from pre to post**.

	**ETG**					**NTG**				
	**Pre median**	**Post median**	**W**	***p*-value**	**Effect-size r**	**Pre**	**Post**	**W**	***p*-value**	**Effect-size r**
**AEROBIC FITNESS AND SENSORIMOTOR PERFORMANCE**										
Step test (Index)	10.60	8.05	−1.363	0.173	0.36	7.50	10.00	−0.943	0.345	0.24
Upper body muscular endurance test (rep.)	26.00	24.00	−0.236	0.813	0.06	22.00	20.00	−1.473	0.141	0.38
Grip strength test R (kg)	21.32	21.17	0.847	0.397	0.23	27.82	35.00	−0.568	0.570	0.15
Grip strength test L (kg)	21.09	22.33	−0.094	0.925	0.03	27.67	32.67	−0.682	0.496	0.18
Pinch force test R (kg)	6.16	7.40	−2.542	0.011^*^	0.68	7.11	7.60	−1.817	0.069	0.47
Pinch force test L (kg)	6.61	6.95	−2.982	0.003^*^	0.80	6.65	7.33	−1.761	0.078	0.45
Motor reaction time test (cm)	17.00	17.75	−0.045	0.964	0.01	17.00	17.00	−0.483	0.629	0.12
JTT R (s)	4.91	4.62	−3.296	0.001^*^	0.88	4.79	4.68	0.00	1.00	0.00
JTT L (s)	5.91	5.27	−3.107	0.002^*^	0.83	5.34	5.33	−1.477	0.140	0.38
Back scratch test R (cm)	−12.50	−11.00	−1.429	0.153	0.38	−2.00	−4.00	−0.399	0.690	0.10
Back scratch test L (cm)	−16.00	−13.50	−1.102	0.271	0.29	−8.00	−11.00	−0.537	0.591	0.14
COP AP EO (mps)	3.90	3.85	−0.440	0.660	0.12	4.80	5.00	−1.221	0.222	0.32
COP AP EC (mps)	7.05	6.00	−2.010	0.044^*^	0.54	6.60	6.00	−2.786	0.005^*^	0.72
COP ML EO (mps)	2.15	1.80	−0.707	0.480	0.19	1.80	1.80	−0.566	0.572	0.15
COP ML EC (mps)	2.90	2.10	−1.992	0.046^*^	0.53	2.80	2.00	−2.328	0.020^*^	0.60
Touch-test R (mN)	3.32	3.25	−1.014	0.311	0.27	3.025	3.22	−0.824	0.410	0.21
Touch-test L (mN)	3.22	3.12	−1.400	0.162	0.37	3.16	3.16	−0.398	0.691	0.10
**COGNITION**										
Alertness (ms)	273.75	241.10	−2.291	0.022^*^	0.61	254.00	238.90	−1.647	0.10	0.43
Simple reaction time (ms)	274.55	251.60	−2.040	0.041^*^	0.55	254.90	241.10	−1.022	0.307	0.26
Working memory (correct)	9.00	10.00	−1.101	0.271	0.29	13.00	11.00	−1.630	0.103	0.42
Response inhibition (correct)	19.00	19.00	−1.706	0.088	0.46	19.00	19.00	−0.203	0.839	0.05

*Wilcoxon-Test for two dependent samples, W, Test-statistic for Wilcoxon-test; ETG, Exergame Training Group; NTG, No Training Group; COP, Center of pressure deviation; AP, Anterior-Posterior; ML, medio-lateral; EO, eyes open; EC, eyes closed; Effect-size r: r = |z/√n|; r = 0.01: small effect; r = 0.30: medium effect; r = 0.50: large effect*.

* *indicate significant finding*.

### Practice effects and baseline dependency of exergaming performance

The overall adherence to the training was 97% with 163 out of 168 sessions (14 participants for 6 weeks/2 sessions per week). The ETG did not show significant differences in their level of attention [*t*_(13)_ = −1.94, *p* = 0.075, *d* = 0.47], fatigue [*t*_(13)_ = −2.01, *p* = 0.065, *d* = 0.56] and discomfort [*t*_(13)_ = 0.66, *p* = 0.520, *d* = 0.09] before and after each training session as assessed by paired *t*-tests. On average, participants showed a high motivation to train at the beginning of each session (mean: 8.77 ± 0.84). Participants of the ETG showed significant online practice improvements in six of the performed disciplines. Two of the strength and all coordination dominated disciplines showed significant performance improvements already after the first half of the training period (Wilcoxon signed rank test for one sample, hurdles Mdn middle session: 6.00% improvement, *Z* = 2.551, *p* = 0.011, *r* = 0.68), (javelin throwing Mdn middle session: 14.69% improvement, *Z* = 2.794, *p* = 0.005, *r* = 0.75), (archery Mdn middle session: 23.29% improvement, *Z* = 3.110, *p* = 0.002, *r* = 0.83), (high diving Mdn middle session: 24.68% improvement, *Z* = 2.605, *p* = 0.009, *r* = 0.69), (trampoline Mdn middle session: 7.30% improvement, *Z* = 3.269, *p* = 0.001, *r* = 0.87), (mountain biking Mdn middle session: 19.08% improvement, *Z* = 3.233, *p* = 0.001, *r* = 0.86) while there were no significant in-game improvements in the endurance-based disciplines. For detailed description see Table 6.

**Table 6:** **Online learning effects in gaming performance**.

**Variables**	**% Improvement from FS to**										**% Improvement from MS to LS**				
	**MS**					**LS**					**LS vs. MS**				
	**Median**	**Min–max**	**Z**	***p*-value**	**Effect-size r**	**Median**	**Min–max**	**Z**	***p*-value**	**Effect-size r**	**Median**	**Min–max**	**Z**	***p*-value**	**Effect-size r**
**STRENGTH**															
100 Meter running	6.46	−1.07–16.84	2.132	0.033	0.57	3.00	−18–21	1.572	0.116	0.42	2.00	−21–12	−0.549	0.583	0.15
Hurdles	6.00	−9–39	2.551	0.011	0.68	7.00	−8–33	2.552	0.011	0.68	0.00	−16–6	−0.78	0.937	0.21
Javelin throwing	14.69	−17.56–101.18	2.794	0.005	0.75	19.2	−20.74–109.66	2.62	0.009	0.70	−3.00	−14–25	−0.454	0.650	0.12
**ENDURANCE**															
Freestyle swimming	4.00	−8–35	1.572	0.116	0.42	1.00	−7–26	0.863	0.388	0.23	−2.00	−13–8	−1.782	0.075	0.48
Butterfly swimming	0.55	−5.48–4.65	1.036	0.300	0.28	2.36	−11.18–6.37	1.852	0.064	0.49	0.79	−12.71–6.48	1.161	0.245	0.31
Hammer throwing	2.78	−6.29–23.60	1.664	0.096	0.44	6.00	−4–19.00	2.132	0.033	0.57	−2.00	−5–4	−1.363	0.73	0.36
**COORDINATION**															
Archery	23.29	−8.52–40.10	3.110	0.002	0.83	32	−7–48	3.040	0.002	0.81	−2.00	−11–6	−2.213	0.021	0.59
High diving	24.68	−32.93–68.45	2.605	0.009	0.69	35	−38–149	2.621	0.009	0.70	−7.00	−48– 25	−0.943	−0.345	0.25
Trampoline	7.30	2.15–43.95	3.269	0.001	0.87	8	3–46	3.180	0.001	0.85	−1.00	−4–1	−2.621	0.009	0.70
Mountain biking	19.08	−2.10–48.82	3.233	0.001	0.86	22.00	12–51	3.180	0.001	0.85	5.00	−9–16	1.992	0.046	0.53

*FS, First Session; MS, Middle Session; LS, Last Session. All disciplines were trained over 12 sessions, except butterfly and freestyle swimming, as well as hurdles and 100 meter running which were trained only over 6 weeks. For those disciplines the scores for the MS therefore represent the performance from the 4th session. Scores from the LS represent the performance from the 6th session. For the remaining disciplines MS refers to the score from 6th session and LS to the 12th session. All values are given in percentage. After Bonferroni correction, a p-value of p < 0.017 was considered as significant*.

## Discussion

The main objective of the current study was to investigate whether an exergame training is able to induce changes in cognitive, as well as sensorimotor performance in healthy older adults. Recent exergame studies addressed this question by using rather long training periods and specific tailor-made training regimes. Hence, our main aim was to elaborate effects based on a multi-modal video game combining endurance, coordination, strength and cognitive demands after a short training period of 6 weeks. We observed significant exergaming induced improvements in fine motor skills of the left hand when comparing performance gains between ETG and NTG. Participants in the intervention group showed significant improvements in online gaming performance across the training sessions for most of the strength and all of the coordination based disciplines. However, these online training effects did not seem to translate into overall performance improvements on the assessed cognitive and sensorimotor functions. While we observed significant improvements in the trained exergame (mainly in tasks that required a high load of coordinative abilities), these gains did not result in differential performance improvements when comparing ETG and NTG.

In an exploratory analysis, within-group comparison revealed improvements in sensorimotor and cognitive tasks (ETG) while NTG only showed an improvement in a static balance test. Taken together, the present study indicates that even though exergames might improve gaming performance, our behavioral assessment was probably not sensitive enough to capture exergaming-induced improvements. To which amount different training parameters such as duration, frequency, or study population contribute to the absence of effect regarding cognition and parameters of motor activity, can not be tested with the given study design. However, different aspects will be discussed in the next sections.

To date, the beneficial effects of exergames on fine motor skills have primarily been shown in clinical studies (Sin and Lee, 2013; Pietrzak et al., 2014; da Silva Ribeiro et al., 2015; Wittmann et al., 2016). For example, Paquin et al. (2015) could show that a 15 min Wii-based training over 8 weeks enhanced upper limb fine motor performance in stroke patients, as assessed by the JTT which was also used in the present study. In a study by McNulty et al. (2011), stroke patients performed a Wii-based training over 10 consecutive days and improved significantly in the Wolf Motor Function Test (WMFT). They concluded that improvements in Wii gaming skills might have generalized to more functional tasks as assessed by the WMFT. The WMFT includes several functional and timed tasks to quantify upper extremity motor ability and is therefore similar to the JTT (Jebsen et al., 1969; Wolf et al., 2001). In the present study, the ETG was able to complete the JTT significantly faster after participating in an exergame training over 6 weeks compared to the NTG, at least in left hand performance. Except from video game training interventions, other lifestyle interventions have already been shown to be effective in enhancing motor performance in healthy older adults (Van Roie et al., 2010; Wu et al., 2010; Kattenstroth et al., 2013; van het Reve and de Bruin, 2014; Eggenberger et al., 2015, 2016). For example, Kattenstroth et al. (2013) performed a 6-month dance intervention in healthy older adults and found beneficial effects in hand-arm steadiness, control precision, and wrist movements compared to a passive control group. Comparable to dancing, playing exergames is based on complex upper body movements and it is therefore assumable that exergaming would also translate into enhanced fine motor skills. The exergame training used in the present study required an intensive use of upper limbs for performing strength and endurance dominated disciplines as well as fine adjusted movements of upper extremity inter-limb coordination. Hence, our findings of improved fine motor performance after 6 weeks of exergame training could be interpreted as transfer of newly acquired motor skills to functional tasks which were not directly practiced during exergaming. Interestingly, exergame induced effects were only visible in significant performance improvements of the non-dominant left hand. This transfer of bilateral training to unilateral improvements has already been well documented in previous studies (Schulze et al., 2002; Burgess et al., 2007; McCombe Waller and Whitall, 2008; Hinder et al., 2013). A study by Schulze et al. (2002) revealed that a bimanual training of the pegboard task leads to improvements in unimanual performance with, however, no difference for the left or right hand. Middleton et al. (2013) demonstrated that after a Wii-based training over 2 weeks young participants performed significantly better in a bimanual and eye-hand coordination task simulating surgical procedures using their non-dominant left hand. One could speculate that task characteristics might explain this effect since performing surgery-like movements require complex functional skills and are therefore visuo-motor and cognitively more demanding compared to a simple motor task. In older adults it has been shown that the performance of unimanual and bimanual tasks requires more neural resources compared to young participants which is known as neural “overactivation” (Mattay et al., 2002; Ward, 2006; Heuninckx et al., 2008; Goble et al., 2010). In a study by Goble et al. (2010), it was shown that older adults exhibited elaborated brain activity in several motor and frontal brain regions during a wrist coordination task compared to younger adults. Moreover, the elaborated brain activation in the supplementary motor area and the somatosensory cortex was positively correlated with higher coordination task demands during antiphasic movements and can therefore be considered a marker for increased task complexity. Hinder et al. (2013) demonstrated a strong transfer of bimanual and unimanual contexts for young and old adults with, however, a release of intracortical inhibition only by older participants. During the exergame training, participants had to perform various bimanual movements. Thus, one could argue that a more elaborated bihemispheric activation while performing the required movements during exergaming might explain the significant improvements in the JTT for the left hand. Nevertheless, the absence of effects in the dominant hand might also be due to an already relatively high skill level compared to the less used hand.

Moreover, Kattenstroth et al. (2013) attributed the enhanced effects in fine motor skills not only to increased sensorimotor coordination but also to muscle strength. Our results corroborate this assumption by showing, that when comparing the within-group effects of the ETG, there is not only a significant performance improvement of fine motor skills of the hand, but also in pinch force of both hands, after 6 weeks of exergame training.

However, since a direct comparison between ETG and NTG did not reach significance, our within-group findings need to be interpreted with caution since sample size might be to small to reach significant between-group effects. The impact on strength by exergame training has been reported mainly in clinical studies with mixed results (Yavuzer et al., 2008; McNulty et al., 2011; Lee, 2013; Sin and Lee, 2013; Laver et al., 2015). In fact, Lee (2013) reported that after 6 weeks of exergame training stroke patients improved in upper body muscle strength which were, however, not significantly different to a control group who received occupational therapy. Up to date, only a few studies were examining exergame trainings using healthy populations with preliminary results indicating that video game training can promote lower limb muscle strength in middle aged woman (Nitz et al., 2010) and older adults (Jorgensen et al., 2013). However, since we only assessed muscle strength of the upper limbs, this assessment might have been not specific enough to capture differential training effects between the ETG and NTG.

The investigated exergame training had no differential effects on aerobic fitness when comparing ETG with NTG. This is in contrast to Maillot et al. (2012) where older adults exhibited improvements in aerobic fitness assessed by a reduction in maximum and mean heart rate in a 6-min walking test after 12 weeks of Wii-based training. While Maillot et al. (2012) were using more stationary games without the necessity of leaving the ground with one foot, in the present study, sport disciplines which required pronounced efforts in running (100 meter running) or walking on the spot while performing additional arm movements (javelin throwing, freestyle swimming, hammer throwing) were given.

Effects of rather classical aerobic training regimes have been intensively investigated within the last years, with results indicating strong associations between cardio-respiratory fitness and performance in cognition (Kramer et al., 1999; Colcombe et al., 2006; Erickson et al., 2011; Leckie et al., 2014). Only a small number of studies have focused on this relationship using exergame training interventions. These studies showed that exergames have beneficial effects on executive functions, processing speed and dual-task-performance which is consistent with findings on aerobic training (Maillot et al., 2012; Schoene et al., 2013). Interestingly, in our study we found no significant between-group evidence for a transfer of exergaming on response inhibition (in a Go/NoGo task) or on reaction time.

However, when looking at the within-group comparison in only the ETG, we found significant performance improvements from before to after the exergame training in alertness and simple reaction time. Interestingly, this effect was absent in the NTG. Accordingly, one could speculate that a training period of 6 weeks might have been too short for inducing distinct changes in cognition, or that the selected cognitive tests were not sensitive enough for capturing these. On the other hand, sample characteristics might have also crucially contributed. Indeed, while participants of the aforementioned studies were living a sedentary lifestyle, our study sample was characterized by an according to their age, healthy and physical active sample of older adults.

It is well established that poor balance and the risk of falling seems to be associated with executive functions and attention since keeping balance requires the integration of somatosensory informations and is further associated with the control and shift of attentional resources (Woollacott and Shumway-Cook, 2002; Granacher et al., 2008; Yogev-Seligmann et al., 2008; Mirelman et al., 2012; Kearney et al., 2013). Consequently, recent research suggest that an effective balance training should include more complex exercises as well as cognitive demands (Halvarsson et al., 2015). The used exergame sport disciplines required the performance of highly demanding movements covering multiple domains within a cognitively challenging environment. Interestingly, we found significant performance improvements in static balance tasks, after 6 weeks of exergame training, in the ETG as well as in the NTG. This effect was however, only visible in within-group pre to post comparisons and did not reach significance for the differential between-group comparisons.

Studies suggested that static (e.g., in quiet stance) and dynamic postural control (e.g., in perturbation or locomotion) are governed by different neuromuscular mechanisms. In a study by Kang and Dingwell (2006) significant differences and no correlation between dynamic stability properties of walking and standing have been found. Interestingly, while participants in the study of Bisson et al. (2007) improved in functional balance, as assessed by the Community Balance and Mobility Scale, no significant changes in postural sway during quite stance were found. A meta analysis on exergaming for balance training pointed out that instrumental assessments of balance often fails to detect small changes whereas more functional clinical tests are too global. van Diest et al. (2013) therefore concluded that only the usage of both functional and objective-instrumental measures of balance is reliable for capturing effects on balance induced by exergame interventions. In the present study, static balance was administered in an objective-instrumental fashion by using a force plate. As a consequence, the absence of specific training-induced effects on balance performance could be due to the applied test which might have not been able to detect slight improvements of our training participants.

The sense of touch is essential for daily life since sensorimotor performance such as keeping balance or motor hand function depends on afferent tactile informations (Lord et al., 1991; Tremblay et al., 2003). To date, the potential training of tactile performance has been mostly investigated using passive stimulation paradigms such as tactile coactivation where small skin portions of the finger are directly stimulated for a few hours. While studies using this procedure demonstrated that tactile acuity can be restored in older adults (Dinse et al., 2006; Kalisch et al., 2008), the potential of exercise interventions is yet largely unexplored. One study reported that an enriched training environment in the form of dancing activities over 6 month can induce changes in tactile performance in healthy elderly (Kattenstroth et al., 2013). Likewise, exergames provide simultaneous sensorimotor and cognitive demands and can therefore be considered as environmental enrichment. Nevertheless, after 6 weeks of exergame training, the ETG in the present study did not exhibit improvements in tactile performance. Our result is in line with the study of Nitz et al. (2010) which could not find significant changes after 10 weeks of Wii-based training in middle aged woman. Interestingly, it has been proposed that long term practice in Tai Chi, a whole-body sensory-attentional exercise, is associated with superior tactile skills (Kerr et al., 2008, 2016). Hence, it seems that exercise interventions could provoke benefits in tactile performance but a training duration of 6 weeks might have been to short for inducing effects, or the assessment procedure used, was not specific enough to capture training-induced improvements in tacile performance in older adults

### Context interference and timing of post assessments crucial for capturing training-induced effects

In the present study, we especially were interested in whether a multi-domain exergame training is able to foster transfer effects to untrained tasks resulting in significant improvements in a variety of sensorimotor and cognitive assessments. Lustig et al. (2009) reviewed different training types with respect to their effectiveness in improving cognitive functioning in older adults. They pointed out that multi-modal approaches often exhibit broad benefits with, however, relatively small effect sizes. Most of the reviewed multi-modal studies did not include longitudinal follow-up assessments and therefore cannot exclude the possibility that results might have been influenced by interference. The context interference effect suggests that learning of multiple tasks will lead to a poorer initial practice performance but induce superior gains in subsequent retention tests as well as transfer to untrained tasks (Magill and Hall, 1990). Numerous studies investigating simple motor paradigms (Lee and Magill, 1983; Albaret and Thon, 1998; Giuffrida et al., 2002; Sidaway et al., 2016) or more complex real-life motor skills (Bortoli et al., 1992; Hall et al., 1994; Sherwood, 1996; Tsutsui et al., 1998; Babo et al., 2008) concluded that a random compared to a blocked order training of multiple tasks seems to promote learning and transfer more efficiently. For the exergame training used in the present study, participants played different sport disciplines from which each combined different quantities of sensorimotor and cognitive demands. Hence, the training procedure was similar to a random design and we therefore assumed that the exergame training will translate into pronounced gains in the post-tests. After 6 weeks of exergame training, we found improvements in fine motor skill of the left hand in the ETG with, however, no overall enhancements. In general, during exercising various peripheral and central acting physiological processes occur and proceed on different time scales (Adkins et al., 2006). Thus, for capturing training induced changes, timing of post-test assessments seems to be crucial. There are conflicting results regarding the amount of time separating acquisition and retention tests. John dos Santos et al. (2014) confirmed superior performance of a practice group, randomly training a dart throwing task, compared to a block training group, 24 h after the training. However, no significant differences in radial distance from the dart to the inner bull were found 7 and 30 days after training. Contradictory to that, in a study by Pauwels et al. (2014), the interleaved practice group outperformed the block group even 1 week after training in a bimanual tracking task. Moreover, superior performance in retention tests has been found even 2 weeks after training of a throwing task (Granda Vera and Montilla, 2003). According to Lee and Magill (1983) better results in retention following interleaved practice can be explained by higher cognitive demands during the acquisition phase since action plans have to be reconstructed constantly. In the present study, post-tests in motor performance measures were administered 7 days after finishing the exergame training intervention. Despite the fact that we did not have a control group for comparing contextual practice effects, one could speculate that interference between the single tasks took place mediated by a information processing overload. Accordingly, post-test measurements were probably administered too late or too early for capturing overall changes in the sensorimotor and cognitive variables.

### Limitations

As a consequence, one limitation of the present study is that we did not examine performance changes in retention tests 2 weeks after training completion. Therefore, it is not possible to draw conclusions about the persistence as well as about the dynamics of behavioral benefits induced by the exergame training. Hence, to draw a comprehensive picture, future studies should include long term retention tests. Furthermore, we did not enroll additional comparison groups to distinguish the domains which were potentially driving the improvements. With the used passive control group, we have accounted for test-retest effects. When investigating effects on motor and cognitive outcome variables manipulated by exergaming, future studies should include a physical activity and cognitive training group for controlling the contribution of each of those domains. Hall et al. (2012) reviewed studies about exergaming in the elderly and consequently emphasized the high potential on physical and mental health outcomes such as enhanced physical mobility, balance, attention, and information processing. Nevertheless, concerns have been addressed regarding methodological issues especially about differences in frequency and duration of interventions as well as the low number of participants. Regarding the duration of physical training interventions, Colcombe and Kramer (2003) stated that a short training period between 1 up to 3 month already showed moderate effects on cognitive function, while longer training periods show substantially larger effects. Accordingly, studies applying the exergame training within this period were able to show significant effects on cognition as well as motor performance (Nitz et al., 2010; Maillot et al., 2012). However, these studies were using participants having a predominantly sedentary lifestyle. In the present study, a sample of active older adults has been used and it is therefore conceivable that the training period of 6 weeks might have been too short for inducing overall effects in active elderly.

However, since we were rather interested in the exploratory investigation on the effects of an commercially available exergame, which is not specificially designed to enhance sensorimotor and cognitive functions, it is possible, that the behavioral improvements only resulted in significant in-game improvements and did not translate into improvements of the specific assessments of sensorimotor and cognitive functions. Finally, for some of our outcome measures (e.g., endurance tests, back scratch test, COP measures), there is no information about the reliability of the respective test available. Hence, our results should be interpreted with caution especially because our study might in fact be underpowered.

## Conclusions

In summary, the present study was the first which addressed the question whether a whole-body exergame training can promote a broad range of sensorimotor and cognitive functions in healthy and active older adults. Interestingly, we observed significant in-game improvements of the exergame training, which was mainly present in disciplines requiring high coordination skills. However, the only exergaming-induced difference was a superior behavioral gain in fine motor skills of the left hand. However, when assessing behavioral improvements of the ETG alone, we saw significant improvements in pinch force, fine motor skills, static balance, and cognitive function, while the assessment of the NTG alone only showed significant improvements in static balance. In conclusion, we found evidence, that 6 weeks of exergame training result in improved gaming performance, but our behavioral assessment was probably not sensitive enough to capture rather global improvements on sensorimotor and cognitive function in older adults.

Therefore, future studies should control for potential driving variables with simultaneous and careful consideration of sample characteristics. Additionally, more knowledge is needed about the underlying neuronal adaptations induced by exergaming which could be of high relevance in preventing pathological age-related brain alterations. Future studies should therefore include neuroimaging assessments in order to identify key regions and significant mental processes acting in multi-domain exergames.

## Author contributions

MO, AV, and PR designed the study. MH and EK helped during data acquisition. MO analyzed the data. MO, MH, EK, and PR wrote the manuscript. All authors were involved in discussing data.

## Funding

MO received a PhD student stipend from the Max Planck International Research Network on Aging (MaxNetAging).

### Conflict of interest statement

The authors declare that the research was conducted in the absence of any commercial or financial relationships that could be construed as a potential conflict of interest.
